# Sensing deep extreme environments: the receptor cell types, brain centers, and multi-layer neural packaging of hydrothermal vent endemic worms

**DOI:** 10.1186/s12983-014-0082-9

**Published:** 2014-11-18

**Authors:** Shuichi Shigeno, Atsushi Ogura, Tsukasa Mori, Haruhiko Toyohara, Takao Yoshida, Shinji Tsuchida, Katsunori Fujikura

**Affiliations:** Department for Marine Biodiversity Research, Japan Agency for Marine-Earth Science and Technology, 2-15 Natsushima-cho, Yokosuka, 237-0061, Kanagawa Japan; Nagahama Institute of Bio-Science and Technology, Institute of Bio-Science and Technology, 1266 Tamura-Cho, Nagahama, 526-0829, Shiga Japan; Nihon University, 1866 Kameino, Fujisawa, 252-0880, Kanagawa Japan; Division of Applied Biosciences, Kyoto University, Graduate School of Agriculture, Laboratory of Marine Biological Function, Kitashirakawa Oiwake-cho, Sakyo-ku, Kyoto, 606-8602 Japan

**Keywords:** Deep-sea, Sensory cells, Brain, Nervous system, Glia, Annelids, Evolution

## Abstract

**Introduction:**

Deep-sea alvinellid worm species endemic to hydrothermal vents, such as *Alvinella* and *Paralvinella,* are considered to be among the most thermotolerant animals known with their adaptability to toxic heavy metals, and tolerance of highly reductive and oxidative stressful environments. Despite the number of recent studies focused on their overall transcriptomic, proteomic, and metabolic stabilities, little is known regarding their sensory receptor cells and electrically active neuro-processing centers, and how these can tolerate and function in such harsh conditions.

**Results:**

We examined the extra- and intracellular organizations of the epidermal ciliated sensory cells and their higher centers in the central nervous system through immunocytochemical, ultrastructural, and neurotracing analyses. We observed that these cells were rich in mitochondria and possessed many electron-dense granules, and identified specialized glial cells and serial myelin-like repeats in the head sensory systems of *Paralvinella hessleri*. Additionally, we identified the major epidermal sensory pathways, in which a pair of distinct mushroom bodies-like or small interneuron clusters was observed. These sensory learning and memory systems are commonly found in insects and annelids, but the alvinellid inputs are unlikely derived from the sensory ciliary cells of the dorsal head regions.

**Conclusions:**

Our evidence provides insight into the cellular and system-wide adaptive structure used to sense, process, and combat the deep-sea hydrothermal vent environment. The alvinellid sensory cells exhibit characteristics of annelid ciliary types, and among the most unique features were the head sensory inputs and structure of the neural cell bodies of the brain, which were surrounded by multiple membranes. We speculated that such enhanced protection is required for the production of normal electrical signals, and to avoid the breakdown of the membrane surrounding metabolically fragile neurons from oxidative stress. Such pivotal acquisition is not broadly found in the all body parts, suggesting the head sensory inputs are specific, and these heterogenetic protection mechanisms may be present in alvinellid worms.

**Electronic supplementary material:**

The online version of this article (doi:10.1186/s12983-014-0082-9) contains supplementary material, which is available to authorized users.

## Introduction

The alvinellid worms are annelids that are generally found on microbial mats closely inhabiting the smokers extruding from the active chimneys of deep-sea hydrothermal vents [1,2]. The fauna inhabiting these hot spring fields are exposed to highly fluctuating physico-chemical conditions, high levels of heavy metals, sulfide, and carbon dioxide, and harmful compounds such as hydrogen peroxide and hydroxyl radicals [3,4]. The emblematic characteristic of these alvinellids is thus their exceptional tolerance to high temperatures and the toxicity of acidic and reducing fluids [5-8]. Indeed, the alvinellid thermostabilization, detoxification, and anti-oxidative stress capacities have been attributed to a number of biochemical, physiological, and structural properties [4,9-13], supported by deep sequencing analysis of the transcriptomic and proteomic level stability [14-16].

Despite these extensive studies, little attention has been given to the sensory and nervous systems, particularly their behavioral ecology. Studies of sensory systems of animals endemic to hydrothermal vents are important for two main reasons. First, the sensory ecology of vent-endemic species is largely unknown, with the exception of some classic pioneering work on alvinellid larval settling [17], and crustacean vision and olfaction (e.g., [18-21]). Second, the neural cells are expected to be highly sensitive to toxic and redox fluids, since the neuronal cells primarily carry out electronic and active chemical signal transduction via synapses (e.g., [22]); therefore, the sensory receptors and neural tissues may possess specific tolerance mechanisms.

Classic anatomical studies have revealed that the overall body, tissue, and cellular organization of *Alvinella pompejana* exhibits a typical polychaete ground plan, without visual and gravity sense organs [23]. The sensory receptor cells identified on the branchial crown and feeding appendages are ciliate cells, mitochondria-rich, and of the bipolar type with single long axon, often associated with supportive cells [24,25]. Thus far the nuchal organs, usually a paired epidermal ciliary structure in most polychaetes [26], have not been found in the prostomium of alvinellids. The structure of the central nervous system has been extensively studied in polychaetes and other annelid taxa [27-31], including a few chemosynthetic siboglinid species [32-34]; however, the ultrastructural organization of the brain and nerves, as well as regional specialization, including inter- and intra-lobe connective patterns, is largely unknown in the deepsea annelids, including terebelliform alvinellids.

Since the first discovery of alvinellids on the East Pacific Rise [23], 11 species have been described in the order Terebellida and family Alvinellidae, which comprises two genera, *Alvinella* and *Paralvinella* [35]. Among the alvinellid species, we examined *Paralvinella hessleri,* which is abundant in the hydrothermal communities of active chimneys. These chimneys can be easily accessed with remotely operated vehicles in the fields of the Izu-Ogasawara Arc or the Okinawa Trough of Japan. Compared to the well-studied *A. pompejana*, *P. hessleri* is smaller in size (total 10 mm or less), thus more individuals can be maintained in restricted laboratory space, and examined with whole-mount, three-dimensional (3D) analysis without dissection. In this study, the detailed physiology of *P. hessleri,* including mechanisms of thermotolerance, were not examined, but our preliminary experiments showed that the thermotolerance of these species is similar to those of *A. pompejana* and *Paralvinella sulfincola* from the North Pacific; *P. hessleri* prefers temperatures between 40-50°C, and endure temperatures as high as 55°C ([15,36]; see also [16]; Shigeno et al., unpublished). Using this species, we sought to provide the first comprehensive maps of the distribution of ciliated sensory cells, neural projections in the higher brain centers, and newly identified cellular components, to explain mechanisms for tolerating the hydrothermal vent environment.

## Results

### Alvinellid body plans

**Figure 1:**
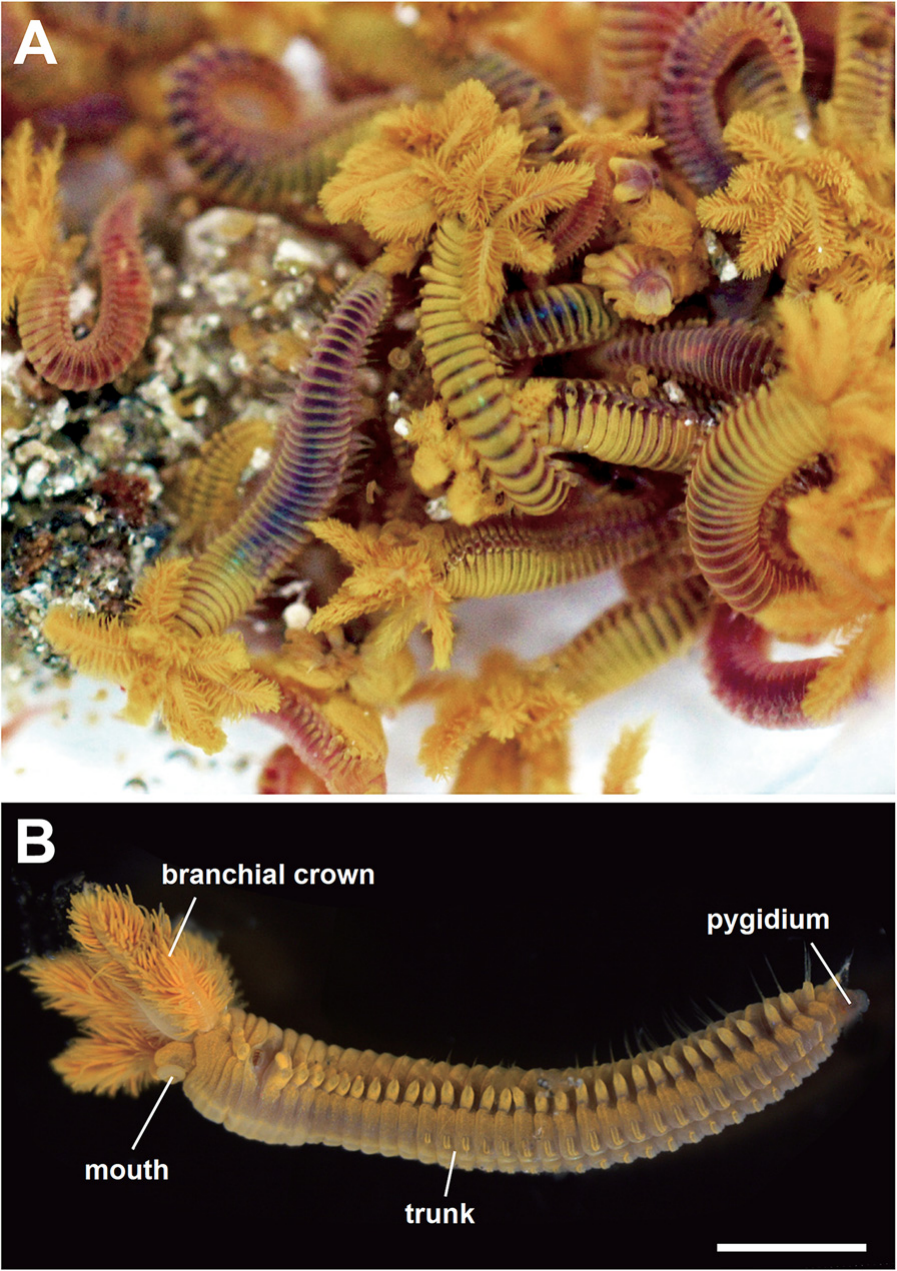
**Images of the vent endemic alvinellid worm,** ***Paralvinella hessleri*** **. A**: Live worms viewed on board. **B**: A paraformaldehyde-fixed sample viewed from the lateral side. Scale bar, 1 mm.

**Figure 2:**
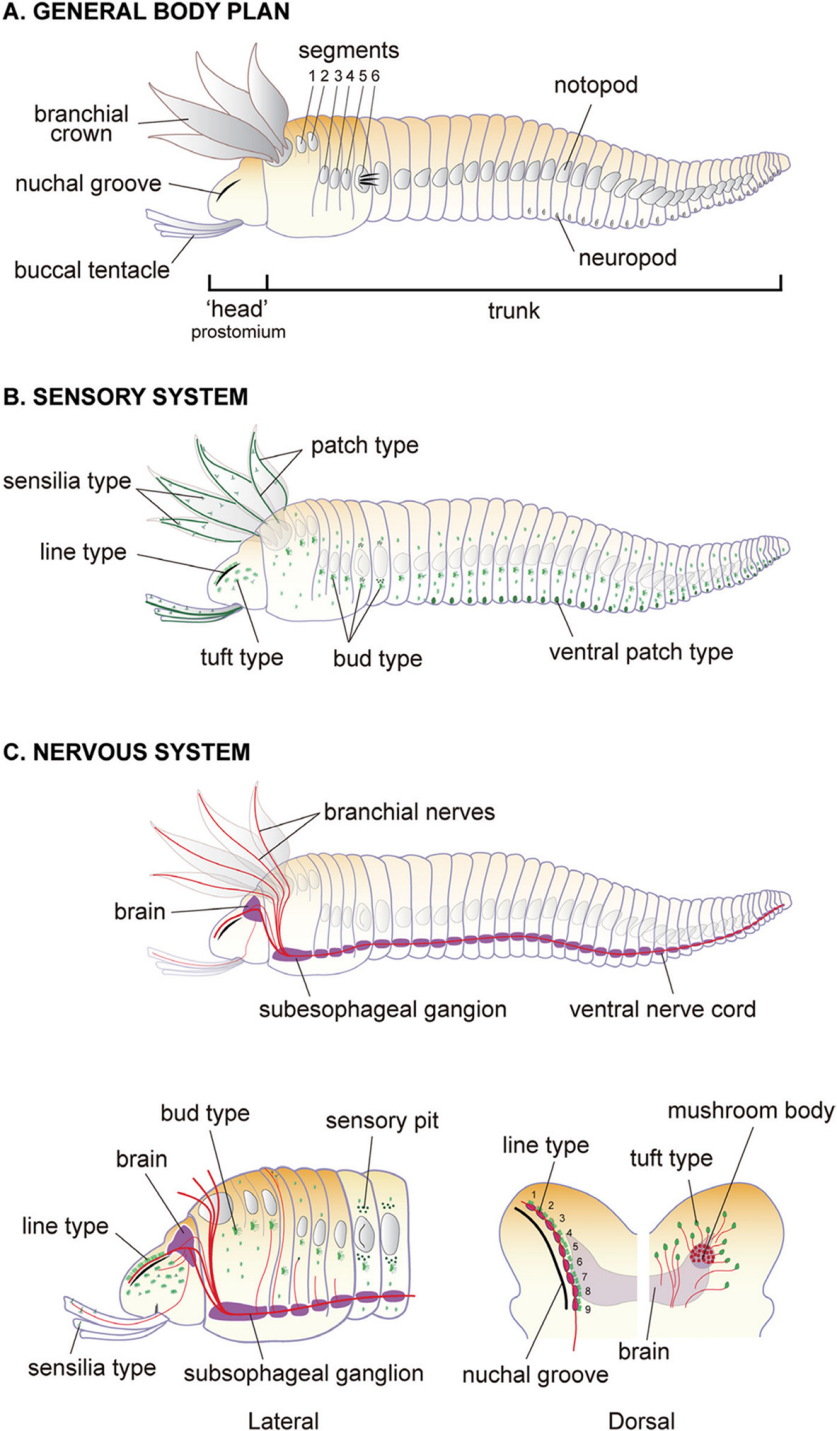
**Schemes of body plans of** ***Paralvinella hessleri*** **: the sensory and neural structure. A**: Lateral view of the general body plan. Most of the parapodia setae are omitted. **B**: The epidermal sensory systems (green), major epidermal cilia. The patch type cilia on the branchial crown are represented as lines, and most of the cilia on trunk are omitted for clarity. **C**: The central nervous system and some peripheral nerves. The cell bodies (purple), major neural axons (red lines), and sensory organs (green patches). Divisions between the subesophageal ganglia and ventral nerve cords are not clear. Below left: The enlargement of rostral parts of the body to show the details of neural projections and epidermal ciliary sensory cells, side view. Below right: Dorsal view of the bi-lobed prostomium and the brain. On the left-hand side, the repeated features of glial and ciliary sensory cells (green) are located inside of the nuchal groove. The position of small globuli cell clusters or the mushroom body and distributed sensory cells are shown with axonal projection to the brain.

The external cylindrical body of *Paralvinella hessleri* is divided into three parts, as defined in most species also known as polychaetes: a head or prostomium, a segmented trunk with many appendages, and a terminal pygidium (Figures 1 and 2; [2,37]). The head part includes a protrusible head-end with a divided shield, and numerous buccal tentacles extending from the rostro-ventral mouth opening. The body wall consists of longitudinal, circular, and oblique body wall musculature used in locomotion. The segmented trunk is the widest part of the body. A few polychaete genera have a large branchial crown on the upper side of the head part that is used in feeding and probably also as “feelers” [38,39]. In the Terebellidae, as in many sessile polychaetes, no traces of sensory palps are found [30]. The epidermal sensory cell types and their ciliary organizations identified in this study are summarized in Figure 2B and Table 1. Some neritic burrowing and tube-dwelling polychaetes have various small eyespots and statocysts (tilt and balance sensors), but these were not found in *P. hessleri*. The central nervous system is composed of the supraesophageal mass or brain, subesophageal mass, and the segmented ventral nerve cords (Figure 2C). The head contains the brain. A pair of nuchal grooves is situated in the dorsal head part, as has been observed in other polychaetes. The details of sensory innervation, including the basic organization of the central nervous system, are schematized in Figure 2C.

**Table 1:** **Classification of the sensory and motor ciliated cell types of** ***Paralvinella hessleri***

**Cell types**	**Body localities**	**Projections in CNS**	**Specific characters** ***ciliary length/width***	**Possible homologies**
Sensilla type	branchial crown	anterior VNC	chemical sensory cells	penetrative sensory cells
	whole bodies	VNC, each segment	chemical sensory cells *1-12/0.3μm*	
Line type	dorsal prostomium	anterior VNC	forming lines on the dorsal head *4-13/0.2μm*	neck ciliary bands?
Tuft type	whole prostomium	brain, MB	round and short cilia *1-3/0.2μm*	penetrative sensory cells
Bud type	ventral area of notopods	VNC	roughly lined with pods *1-21/0.1-0.2μm(sharp)*	olfactory organ-like
Patch type	lateral branch and whole bodies	-	typical motor cilia *1-12/0.2μm*	motor ciliary cells
Ventral patch type	ventral abdomen	-	ventrally localized *1-11/0.2μm*	sensory receptor cells?
				lateral organs

Possible homologies are based on Purschke [38]. *MB*, Mushroom body; *VNC*, Ventral nerve cord.

### The sensory receptor cell types

**Figure 3:**
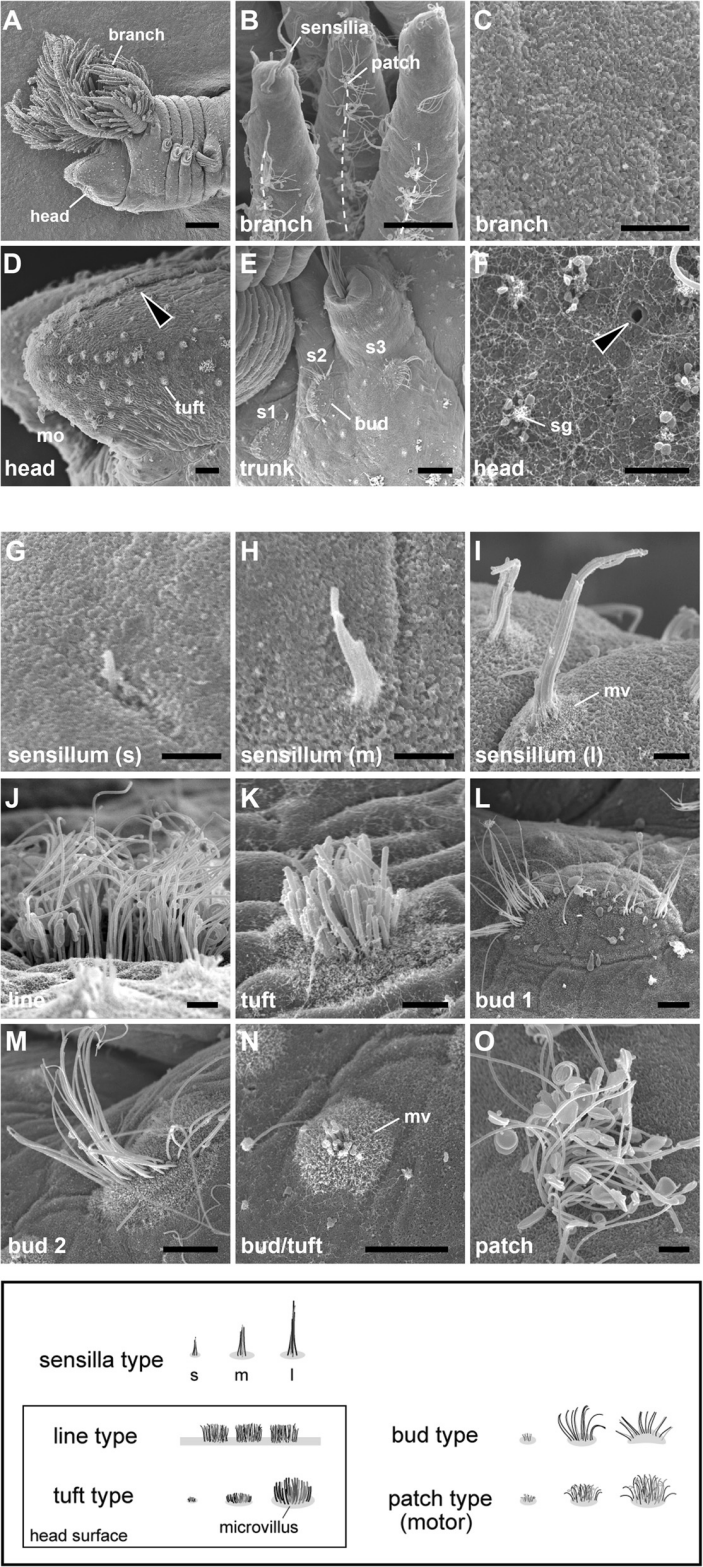
**Scanning electron microscopy images of epidermal ciliary sensory cells.** The cell types are summarized below for each body part. **A**: An overall side view of the rostral part. **B**: Branchial crown and the dotted lines show lines of patch type cilia along the lateral sides. **C**: Enlargement of a branch to show the no-pore condition of the epidermis. **D**: The head part and nuchal groove (arrowhead). **E**: The setigerous segments to show the distribution of the bud type cilia. **F**: The pores (arrowhead) and granules on the epidermis of the head. **G - O**: The epidermal cilia. mo, mouth opening; mv, microvilli; s1-3, setigerous segment 1–3; sg, secretory granules. Scale bar in **A**: 100 μm; **B**, **D**, **E**: 20 μm; **M-O**, 5 μm; **C**, **F**, **G-L**: 2 μm.

We first focused on the main alvinellid epidermal ciliary sensory cells, which are used for chemical and mechanical sensing, as proposed by neritic polychaete studies [38]. The six cell types were identified and categorized, and some subtypes were defined by subtle structural differences of cilia and microvilli (Table 1). The distribution patterns of ciliary cells and pores for excretion are different in each body part; namely, the branchial crown, the head, and the trunk with appendages (Figure 3A-F).

1. **The sensilla type (Figure**3**G-I):** These cells are ciliate sensory cells with a short distal process, also known as typical bipolar receptor cells [38]. Three to five cilia penetrate the cuticle, and the sensory cells send long axons directly to the central nervous system. This cell type is broadly distributed on the head, branches, buccal tentacles, and the trunk. The three (the short, intermediate, and long) ciliary subtypes were detected. The short and intermediate types seem to be developing cell, but here we distinguished as a sensilla type due to their shapes, ciliary numbers, and thickness of cilia.
2. **The line type (Figure**3**J):** These are penetrative multiciliate cells, and the cilia and cell bodies exhibit bilateral longitudinal lines running inside of the dorsal grooves extending from the head end. The ciliary cells are superficially lined, but more detailed analysis identified subtle repeated gaps among these cells. The cilia are generally long and similar in length (10–12 μm).
3. **The tuft type (Figure**3**K):** The cells are multiciliate penetrative type, namely the cilia penetrate the cuticle. The ciliary shape is round and tuft-like. One major subtype is distributed only in the dorsal, lateral, but not in the ventral side of the head. The cilia are short and densely packed compared with those of the patch ciliary type. In contrast to the branch surfaces, many pores and granules are found in the epidermis of the head part (Figure 3F).
4. **The bud type (Figure**3**L-N)**: These penetrative multiciliate cells are situated in their characteristic positions on the head and trunk. As in polychaetes, the bud may correspond to the papilla [30,38]. The cilia are sharp and roughly arranged in a short line.
5. **The patch type (Figure**3**O)**: This multiciliate cell type may have motor functions for producing directed water flow or removing waste matter from the body surface, but chemical or mechanical sensation also may be possible. The axonal projection is lacking and cells are tightly connected to the epidermal cells by the gap junction (not shown). These cells are most dense on the lateral sides of each branch, and are broadly distributed over the entire body. The cilia are not sharp as in the bud type and usually exhibiting loop-like form as a possible artifact (Figure 3O).
6. **The ventral patch type at the trunk (Figure**2**B)**: The ciliary structure is almost identical to the patch ciliary type, with ciliary density and length as stated above. Yet the distribution of cells displays a stereotypic linear pattern, and repeated positions are only observed in the ventral and ventro-lateral sides of the trunk (see the trunk part).

The sensory cells were detected using at least three independent methods: scanning electron microscopy (SEM), transmission electron microscopy (TEM), and light microscopy using labeling for acetylated alpha-tubulin, a widely used cytoskeleton marker for cilia and neural axons (shown later). Phalloidin fluorescent staining was also helpful for detecting F-type actin in the ciliary cells and neurons in differential contrast to the tubulin labeling (not shown).

**Figure 4:**
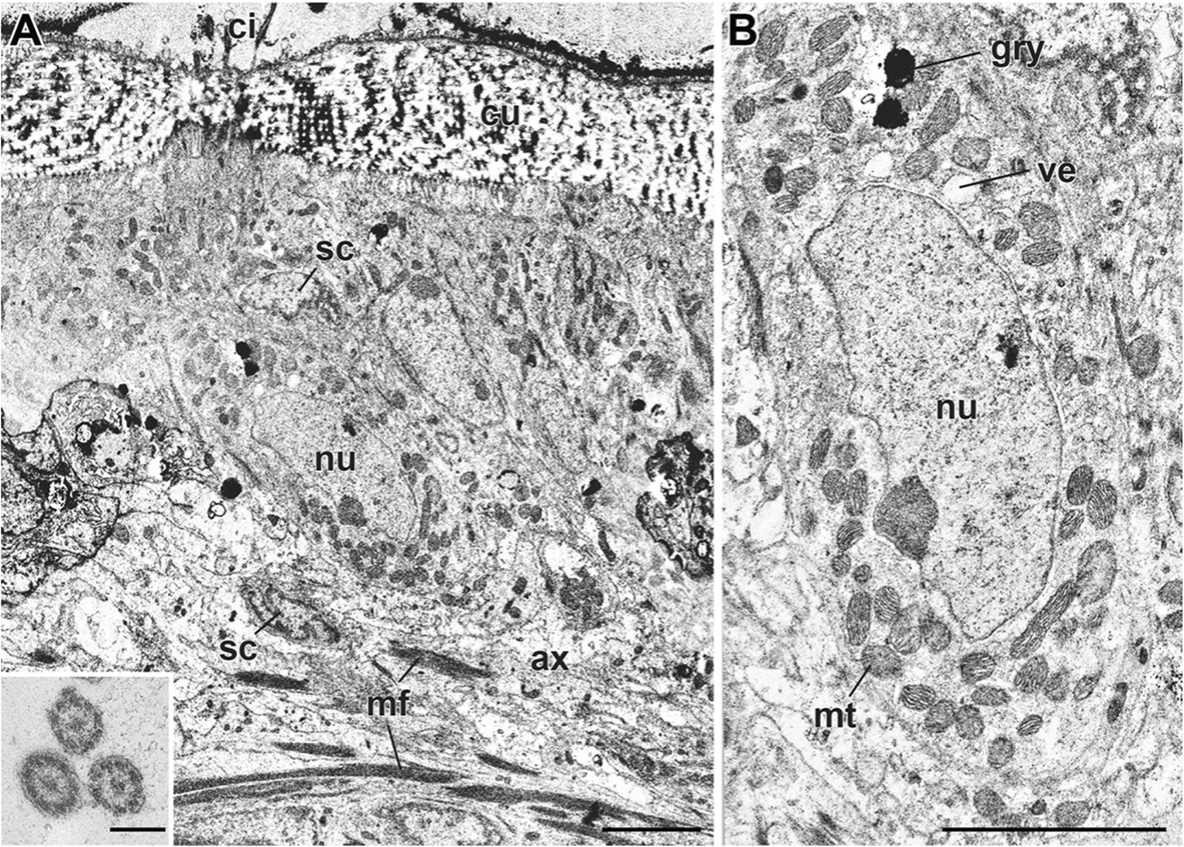
**A primary sensilla cell type and the intracellular organella.** Transmission electron microscopy (TEM) images. **A**: Cross section of the single layer epidermis of the branchial crown. **B**: Enlarged view of the same cell to show the organella. Inset: a cross-section showing the microtubules of cilia outside of the cuticle. ax, axon; cu, cuticle; gry; yellow-color granule; mf, muscle fiber; mt, mitochondrion; nu, nucleus; sc, supporting cells; ve, vesicle. Scale bar in **A**, **B**: 5 μm; Inset, 200 nm.

As revealed with TEM, the typical sensory ciliary cells include multiciliate bipolar types, which are situated in the single epidermal cell layer under the thick cuticle (see Figure 4A for a representative cell). As described previously, the mitochondria structure, including the outer and inner membrane with the cristae and matrix, are typical (Figure 4B; [24]). The size range is from 0.5 to 1.0 μm in diameter. The number of mitochondria per cell varies by tissue type; many epithelial cells, including primary sensory cells, typically have several thousand mitochondria. The electron-dense granules are often observed in the intracellular spaces.

### The myelin-like glial repeats in the head sensory neurons

**Figure 5:**
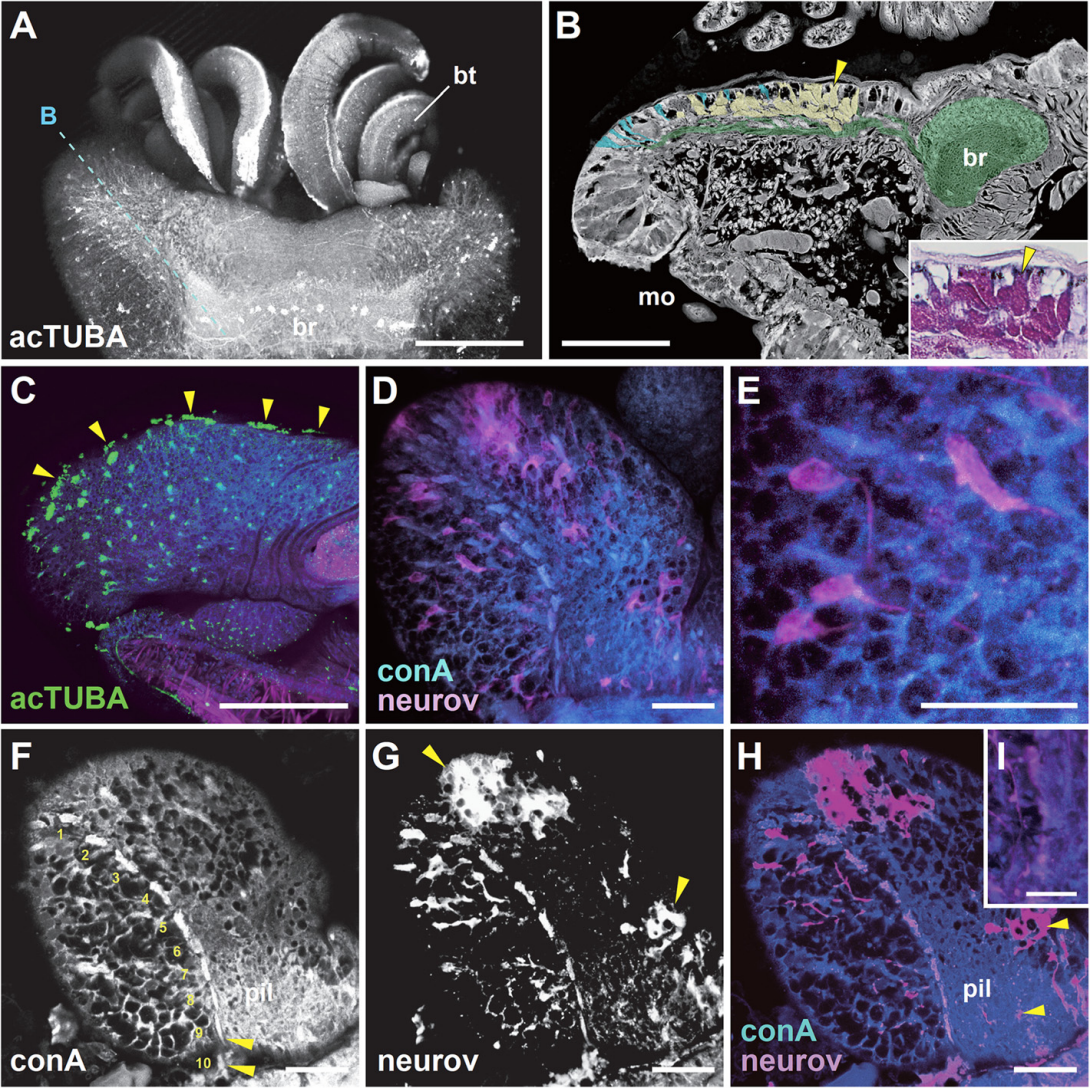
**The brain, ciliary sensory cells, and glial repeats of the head or prostomium: whole-mount laser confocal microscopy images. A**: Dorsal view of the prostomium with buccal tentacles. **B**: A semi-sagittal histological section cut as shown in A, showing a negative image of the hematoxylin and eosin stained section. Pseudocolors were used to enhance the position of line type cilia (light blue), axonal bundles and the brain (green), and secretory cells in the nuchal groove (yellow). Inset: granules stained with eosin. The arrowheads indicate the same position. **C**: A side view of the prostomium showing the distribution of acetylated alpha-tubulin (acTUBA) positive ciliary cells. The arrowheads indicate the line type cilia. The concanavalin A (conA, blue) and phalloidin (actin, red) cell membrane or muscle markers used for counterstaining. **D, E**: The sensory cell bodies and their axons coupled with dye (neurov: NeuroVue®Red) and repeated myelin-like glia stained with conA membrane marker (blue): a dorsal view of whole-mount prostomium and the enlargement. **F-G**: The sensory cells and the axonal projection patterns in the image series of conA, neurov, and merge. The numbers indicate the glial repeats and the arrowheads are positions filled with dye, and some labeled axons in the brain. **I**: Enlarged view showing the axons in the glial repeats. br, brain; bt, buccal tentacle; mo, mouth opening; pil, neuropil. Scale bar in **A-C**: 100 μm; **D-H**: 40 μm; **I**: 20 μm.

**Figure 6:**
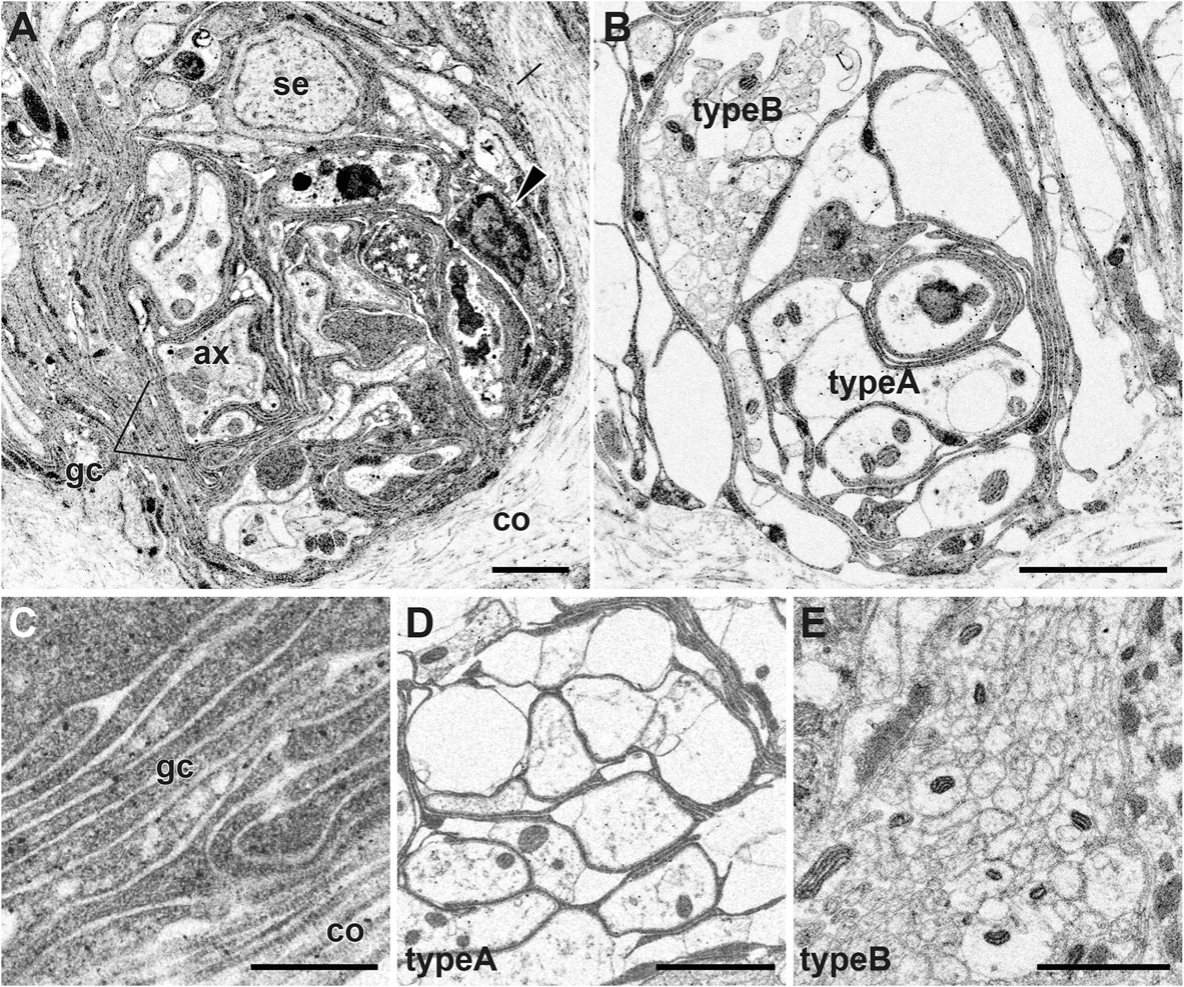
**The myelin-like glial wrappers.** The transverse sections, TEM images. **A**: The axonal bundles cut with sensory cell level, and **B**: the glial inter-repeat region with two different axonal types. The arrowhead indicates possible glial cell body. **C**: The detailed structure of glial membrane. **D**: Two axonal bundles. The type A and B run along the ventral and dorsal sides, respectively. **E**: Type B axonal bundles showing details of the membrane and mitochondria. ax, axonal fibers; co, collagen fibers; gc, glial cell wrappers; se, line type sensory cell. Scale bar in **A**, **B**, **D**: 2 μm; **C**, 300 nm; **E**: 1 μm.

One of the most striking features in the sensory system of *P. hessleri* is the placement of a multiply repeated glial structure for the long axons extending from the longitudinal head ciliary lines at the lateral sides of the granule cell-rich nuchal grooves of the head part. Based on the topographical position of the prostomium or head (Figure 3D shows the surface, and Figure 5A, B show granule cells), the longitudinal ciliary lines can be compared to those of nuchal sensory organs of neritic polychaetes (Figure 5C; [38]), or modifications. In the whole-mount specimens, the lipophilic dye tracer experiments revealed the structure of primary sensory cells spreading over the head surface (Figure 5D, E; Additional file 1: Movie S1), and the axonal fibers derived from the sensory cells of the longitudinal ciliary bands extending from the rostral head ends (Figure 5F-I). At least 10 distinct repeated swellings were morphologically identified using concanavalin A membrane staining (Figure 5F). Their afferents do not directly enter into the brain neuropils, but run along the lateral sides of the brain (Figure 5F). The ultrastructural analyses of the axonal bundles show the position of the glial cell body and multi-layered membranes (about 70–100 nm thickness with a 20 nm gap, Figure 6A-C). In these bundles, we identified differentially arranged types, including loosely organized thick bundles (type A: 1–2 μm ), and tightly organized and fasciculated fine bundles (type B: 0.2–0.6 μm ) (Figure 6D, E).

### Arealization

**Figure 7:**
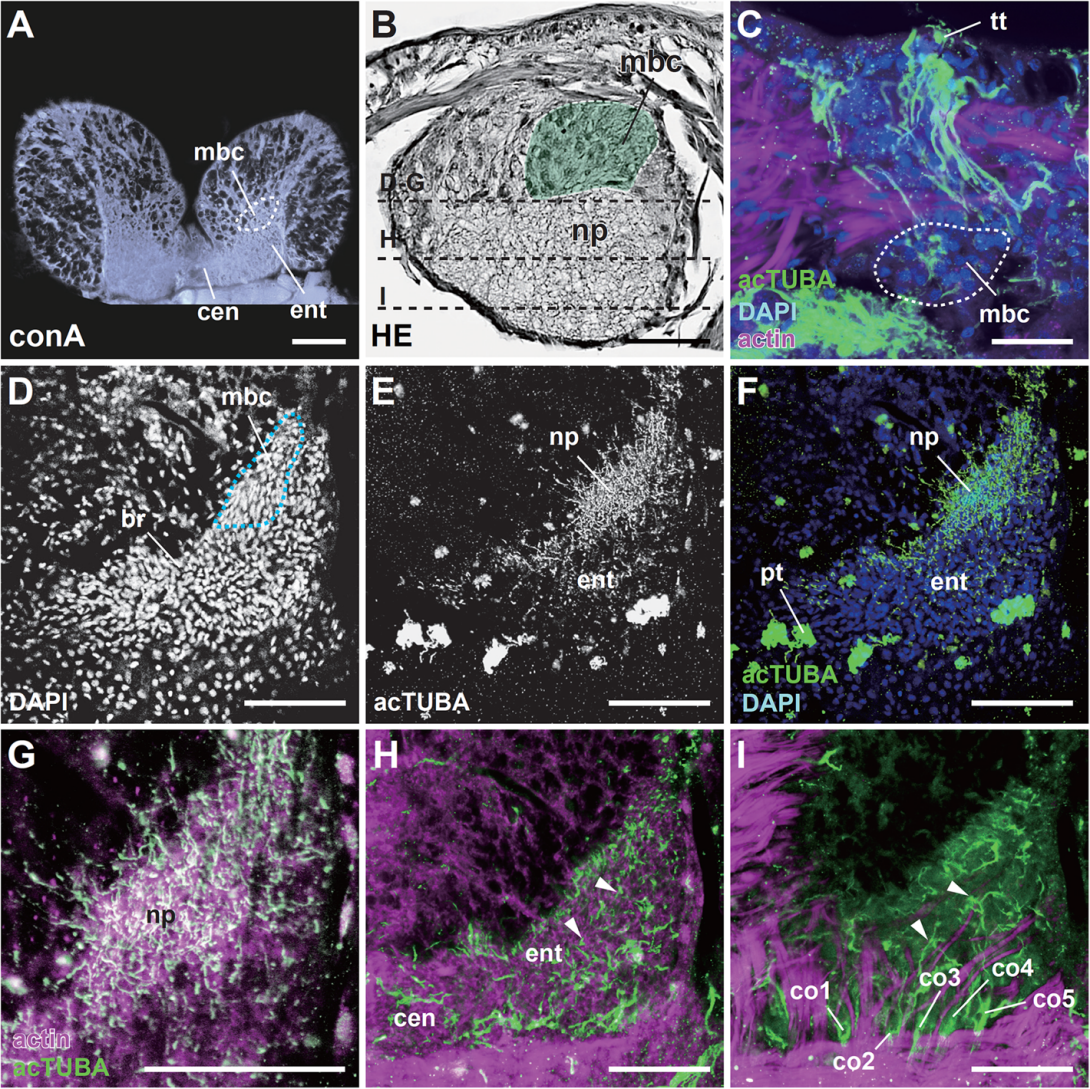
**The brains and higher sensory centers. A**: A horizontal confocal optical section of the whole head region. **B**: A sagittal histological section of the head to show the brain subdivisions. The white dotted line covered with pseudo-green color indicates the position of the mushroom body-like structure in a section stained with hematoxylin-eosin. The dotted lines indicate optical cutting sites for confocal microscopy. **C**: The direct sensory pathways from tuft type cilia to the mushroom body, a sagittal section of the head. **D-F**: The nuclei distribution and neuropil structure. Horizontal optical sections viewed from the dorsal side at the level of the neuropil of the mushroom body (encircled with dotted red line). **G-I**: The highly mingled axons from small mushroom body neurons **(G)**, the brain neuropil **(H)**, and more ventral region **(I)**. Nuclei stained with DAPI and tubulin positive fibers to show the distinct neuropils and axonal bundle patterns. The horizontal optical section series of confocal microscopy viewed from the dorsal (globuli cluster level) to ventral side. Arrowheads indicate the axonal fiber patterns running from dorsal to ventral, and to connectives. The phalloidin (actin) was used for counterstaining of neural fibers and muscles. br, brain; cen, central neuropil; co 1–5, connective to ventral nerve cords; ent, sensory axon entry region of the neuropil; pt, patch type cilia; mbc, mushroom body cells; np, neuropil; tt, tuft type cilia. Scale bar in **A**, **D-I**: 100 μm; **B**: 50 μm; **C**: 20 μm.

**Figure 8:**
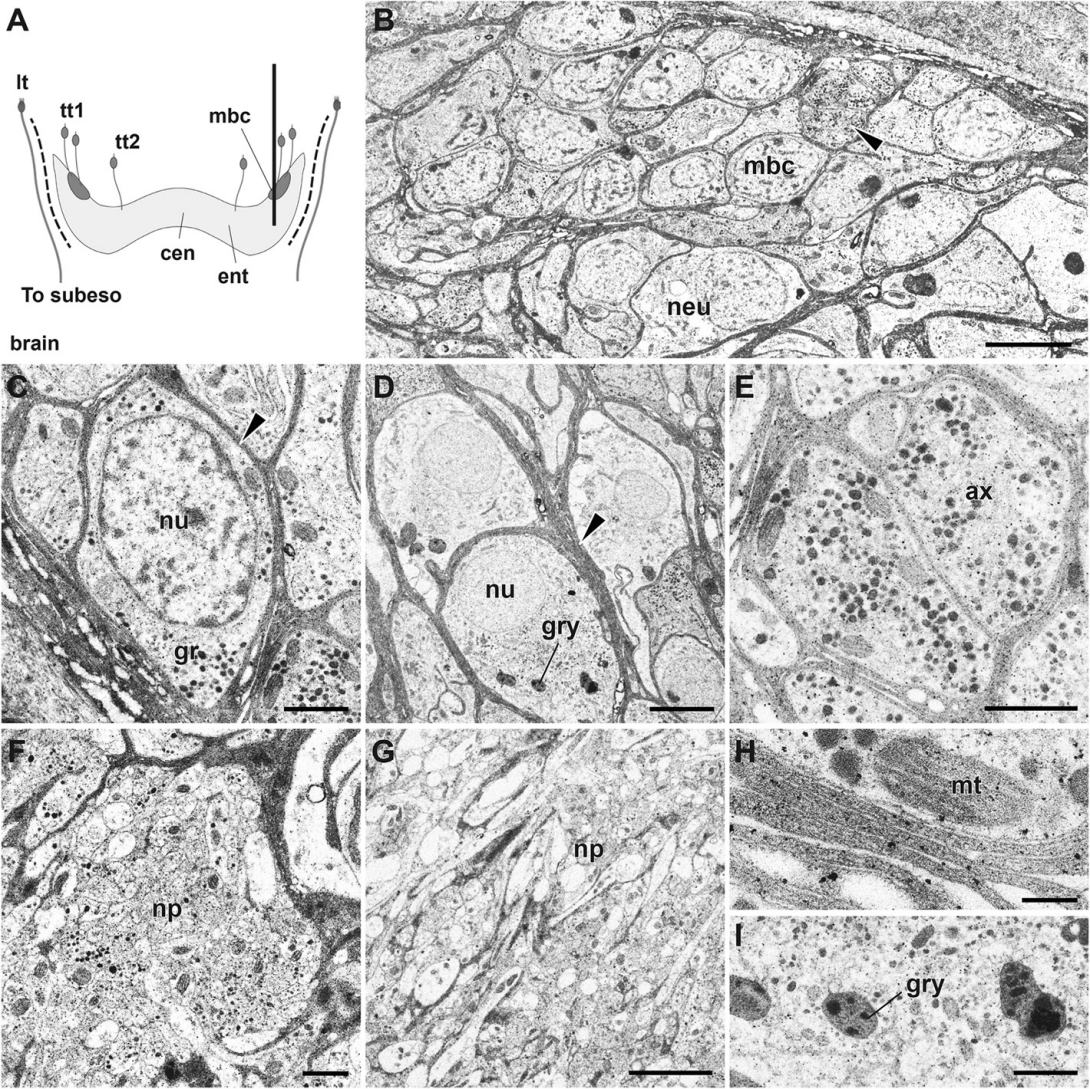
**The mushroom body-like structure and the cell body wrappers.** A schematic figure and TEM images. **A**: Schematic figure showing the brain organization and associated nerves, dorsal view. The thick longitudinal line indicates the cutting site for Figure 8B. **B**: TEM images of small interneurons and a large neighbor neuron. The arrowhead indicates granule-rich cell. **C, D**: The small or large interneurons covered with glia (an arrowhead). **E**: The granule-rich axons from the small cells. **F**: The neuropil of mushroom body. **G**: Details of the mushroom body neuropil. **H**: The enlarged view of glial membranes of small cells. **I**: Granules in the neurons. ax, axons; ent, sensory axon entry region of the neuropil; gr, granules; gry, yellow color granules; lt, line type sensory neurons; mt, mitochondrion; neu, a large neighbor neuron; np, neuropil; nu, nucleus; tt1 an tt2, tuft type cell 1 or 2; mbc, mushroom body cells. Scale bar in **B**, **D**, **G**: 4 μm; **C, E, F**: 1 μm, **H**, **I**: 0.1 μm.

The combined whole-mount immunocytochemistry and the serial optically dissected sections delineated gross features of the brain of *P. hessleri* (Figure 7A, B). The cord-like brain is situated dorsally in the head, and is connected to the subesophageal cords by thick connectives (summarized in Figure 2C). The acetylated alpha-tubulin positive axons and lipophilic neurotracing studies confirmed that most identified sensory inputs from the surface of the head flow into the overall brain neuropils (Figure 7C). In the brain and associated neuropils, three centers are distinct in the dorsal to ventral regions: 1) a tiny pair of mushroom bodies or globuli cell clusters, characterized by small interneuron pools, receive the sensory inputs from the head tuft type cells, and form a small neuropil with numerous fine and less fasciculated axons (Figure 7B-G); 2) the antero-dorsally located entry area of axons of head sensory cells (Figure 7H); and 3) the commissural or central region, situated at the basal region (Figure 7I; Additional file 2: Movie S2). Those regions may be comparable with the brain of some mobile polychaetes such as *Nereis diversicolor;* however, the brain or the brain of the latter contains subdivisions such as the optic neuropil, central neuropil, and more small neuropils [40,41], but such obvious subdivisions are lacking in *P. hessleri* (summarized in Figure 8A).

### The mushroom body-like higher brain centers

The mushroom bodies traditionally called corpora pedunculata have been extensively studied as higher chemosensory learning centers in arthropods and annelids [42,43]. In *P. hessleri*, we found a candidate pair of mushroom body cell clusters in the typical position in the dorso-lateral sides of the brain (Figure 8A), which receive inputs from sensory cells in the dorsal head regions (see Figure 7C). The ultrastructural studies revealed that the small interneurons of the mushroom bodies are histologically distinct compared with the surrounding intermediate-size interneurons (Figure 8B). In the small interneurons, there are a few mitochondria and more than 40 to 80 electronically dense particles (ca. smaller than 100 nm in diameter) in a single ultrathin section (Figure 8C). More characteristically, we found a unique support system for the neural cell bodies covered with glial cells (Figure 8D), and granular-rich axonal bundles in the mushroom body region (Figure 8E), although such glial membranes were not widespread in the neuropils (Figure 8F, G). The spatial distribution of the glial cell bodies could not be precisely determined due to their complexity, but most of neuronal cell bodies are tightly covered with thick membrane comprised of two to three layered glial membranes (Figure 8H). Characteristic fine dense granules are also distributed in some axons and cell bodies (Figure 8E), and may be neurosecretory cells (as described in the brain of other polychaetes [29,44]), but larger granules of a different type are also seen (Figure 8I).

### The sensory systems of the head branchial crown and buccal tentacles

**Figure 9:**
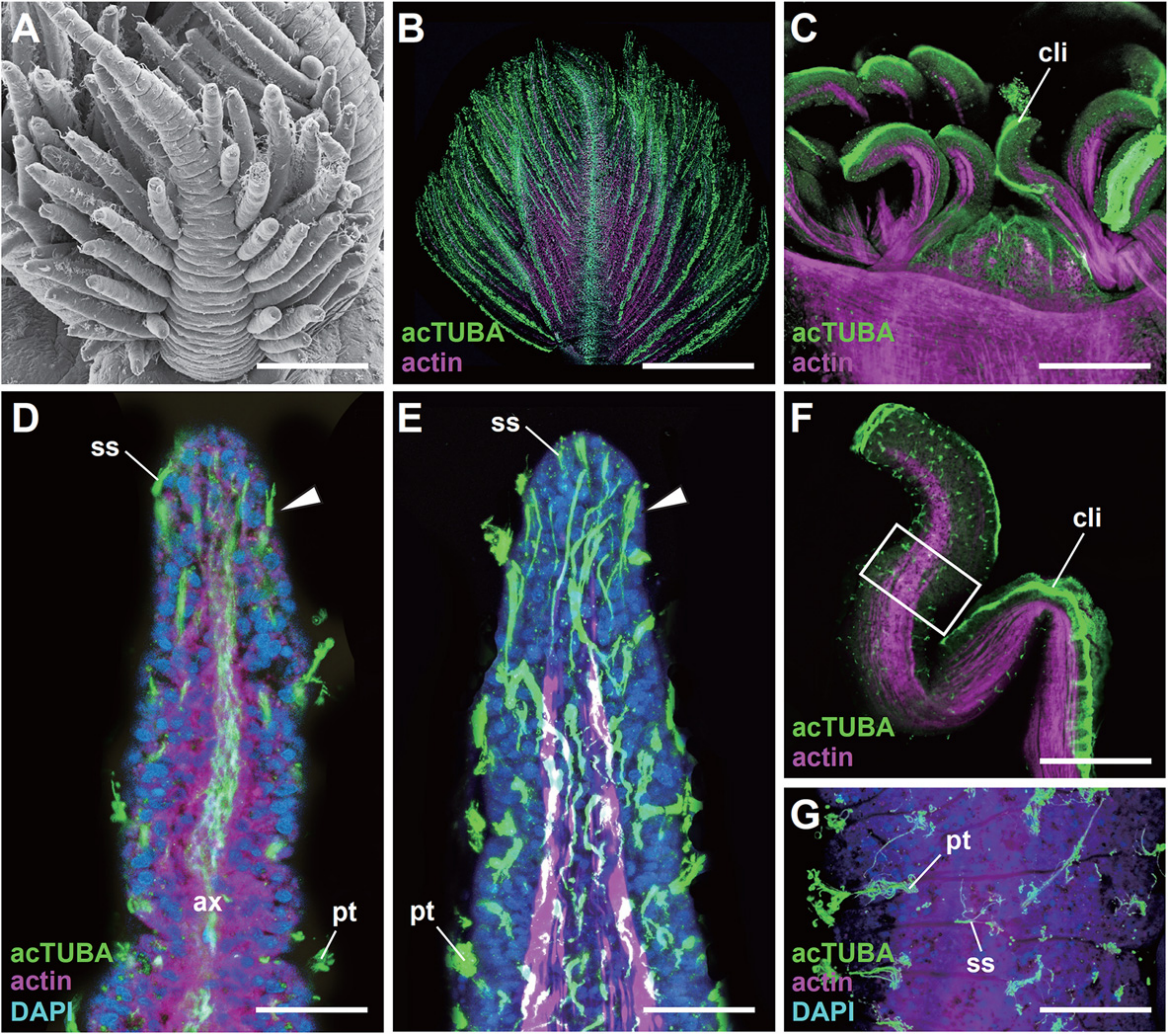
**The ciliary epidermal cells of the branchial crown and buccal tentacles. A**: Scanning electron microcopy image of a branch. **B-G**: Laser confocal microscopy images. **B**: The whole view of the single branch with many patch type cilia. **C**: The buccal tentacles with dense patch type cilia arranged along each ventral side. **D**: An optical section of the leaflet showing the sensillia type cells (arrowhead) and axonal bundles. **E**: The 3D reconstruction images of optical sections showing the pathways from the sensilla type cells. **F, G**: A buccal tentacle and enlarged view to show the distribution of cilia. ax, axon; cli, ciliary line; pt, patch type cilia; ss, sensilla type cell. Scale bar in **A-C**, **F**: 100 μm; **D**, **E**: 10 μm; **G**: 25 μm.

We next focused on the branchial crown (Figure 9A, B) and buccal tentacles (Figure 9C) in the anterior body part. These are important sensory organs for accessing outer fluids and food sources [25], and a previous study showed that the branches exhibit higher anaerobic enzyme activity than abdominal tissues [45].

### The branchial crown

Most of the sensory cells are the primary sensilla type (summarized in Table 1). In each branchial leaflet, there are numerous patch type cells along the lateral sides, and broadly distributed sensilla types (see Figure 3A). Whole-mount immunocytochemistry identifies the ciliary position as well as the axonal origins in the branches (Figure 9D, E; Additional file 3: Movie S3). Notably, we failed to detect the multilayer glial membranes that were found in the head axons in any branchial sensory cells (data not shown).

### The buccal tentacles

The tubulin immunochemistry distinguished the ciliary sensory cell types, and showed that most of the sensory cells are sensilla and patch type cells (Figure 9F, G). As in the case of the branchial crown, the multilayer glial membranes surrounding the axons are not distinguished, as described in previous studies [24].

### The trunk nervous system

**Figure 10:**
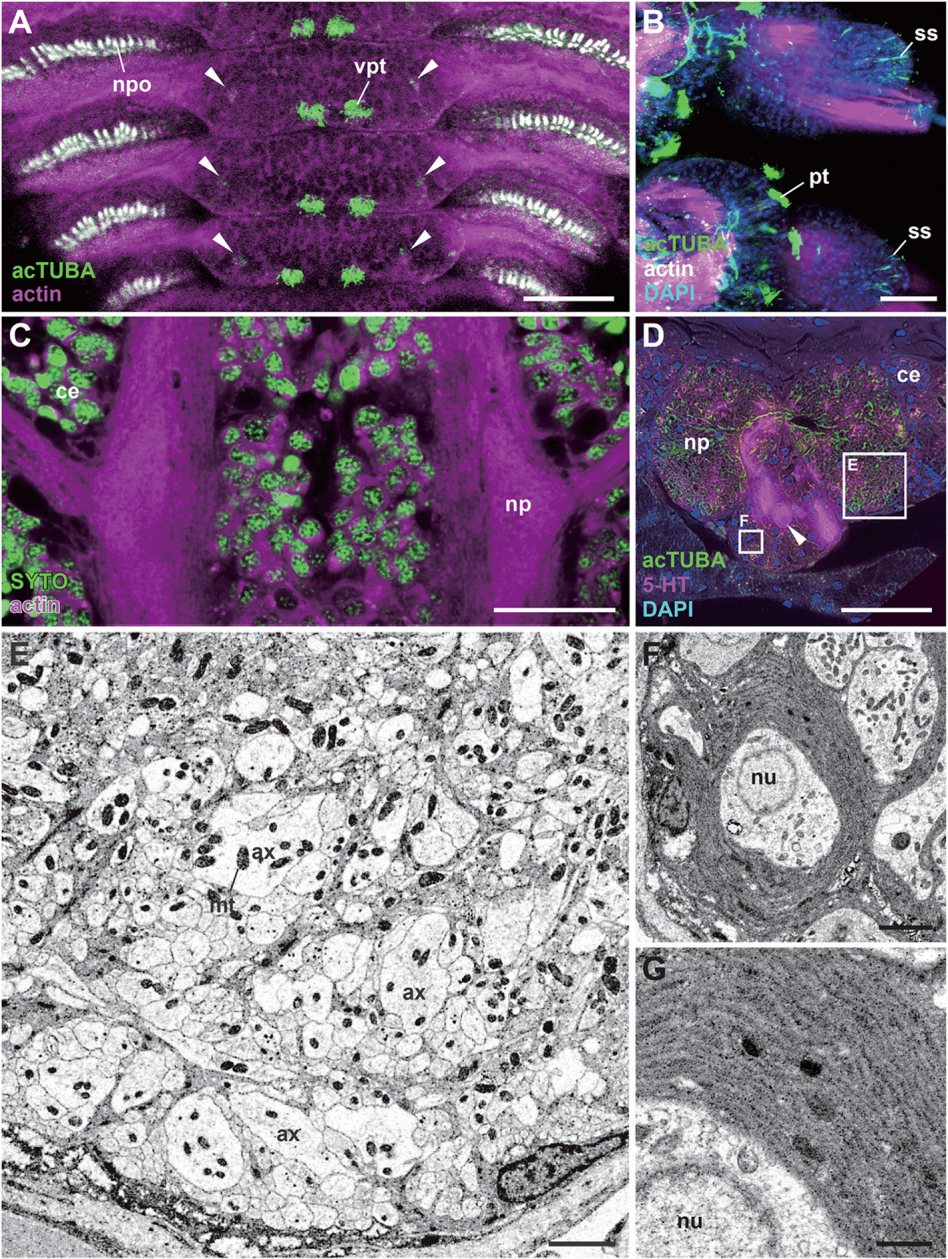
**The trunk nervous system with the sensory cilia and ventral nerve cord.** A-D: The laser scanning microscopy views. **A**: Distribution of ventral patch type cilia with smaller cilia allayed along the ventro-lateral sides (arrowheads) with autofluorescence of the neuropodia. **B**: Notopodia and sensilla distributed at the tips. The axonal projections are seen from sensilla of the tips (ss), but such projections are not seen from patch type cilia (pt). **C**: Cellular distribution and neuropils of the ventral nerve cord. **D**: A cross-section of the ventral nerve cord to show the position of tubulinergic and serotonergic cells (arrowheads) within the cell body layers and axons in the neuropils. The two rectangles indicate the position of analysis for Figures E and F. The TEM images. **E**: Neuropil without obvious wrapping structure. The large axons are possibly motor neurons. **F**: The wrapped cell body of the ventral nerve cords. **G**: An enlarged view of glial membranes. ax, a relatively giant axonal bundle; ce, cell body layer; ss, sensilla type cell; pt, patch type cell; mt, mitochondrion; np, neuropil; nu, nucleus; npo, neruopodium. Scale bar in **A-D**: 50 μm; **E**, **F**: 2 μm; **G**: 0.1μm.

Although we could not detect specific supportive glial systems in the branchial crown and buccal tentacles, such systems might have widely established components in the trunk region, since the myelin—a lipid-rich multilayer sheath surrounding the axons—is found in the ventral nerve cords of some annelid worms [46]. We explored this hypothesis by examining the peripheral nervous systems of the trunk region of alvinellids. Each segment includes a pair of paddle-like parapodia, which consist of setigerous notopods used for locomotory behavior and possibly as sense organs. Many ciliary penetrative sensory cells, including the sensilla, patch, bud, and ventral patch type cilia were found (Figure 10A, B; summarized in Table 1; Additional file 4: Movie S4, Additional file 5). As in most polychaetes, their sensory cells are connected to the ganglionated ventral nerve cords. The giant fibers exist in the typical anterior position of the ventral nerve cords (not shown), but myelinated glial repeats (as observed in the head parts) were not identified in the neuropils by our 3D confocal and ultrastructural analyses (Figure 10C-E). A pair of ventral nerve cords is medially centralized; additionally, most of the neural cells are monopolar, and their axons usually form the neuropils, as revealed by acetylated alpha-tubulin and serotonin-like immunoreactivity (Figure 10D). The neuropils have no obvious compartments by glia (Figure 10E). Some large fibers, possibly longitudinally projected motor axons running along the antero-posterior axis, were detected by the ultrastructural analysis (Figure 10E). The most distinguishing feature is the multi-layered glial membranes in the cell body layer, which are distinct and better developed than in the brain (Figure 10F, G). Nearly all cell bodies examined are covered with glial membranes.

## Discussion

Unique biological systems are often expected to be discovered in animals living in unusual environmental conditions. In order to contribute to the discussion of the sensory and neural systems animals from hydrothermal vent environments, we studied the cellular, intracellular, and histological structure in *Paralvinella hessleri*, a member of the vent-endemic alvinellids, using electron microscopy, immunohistochemistry, and neurotracing in combination with laser confocal microscopy.

### The sensory receptor cell types

**Figure 11:**
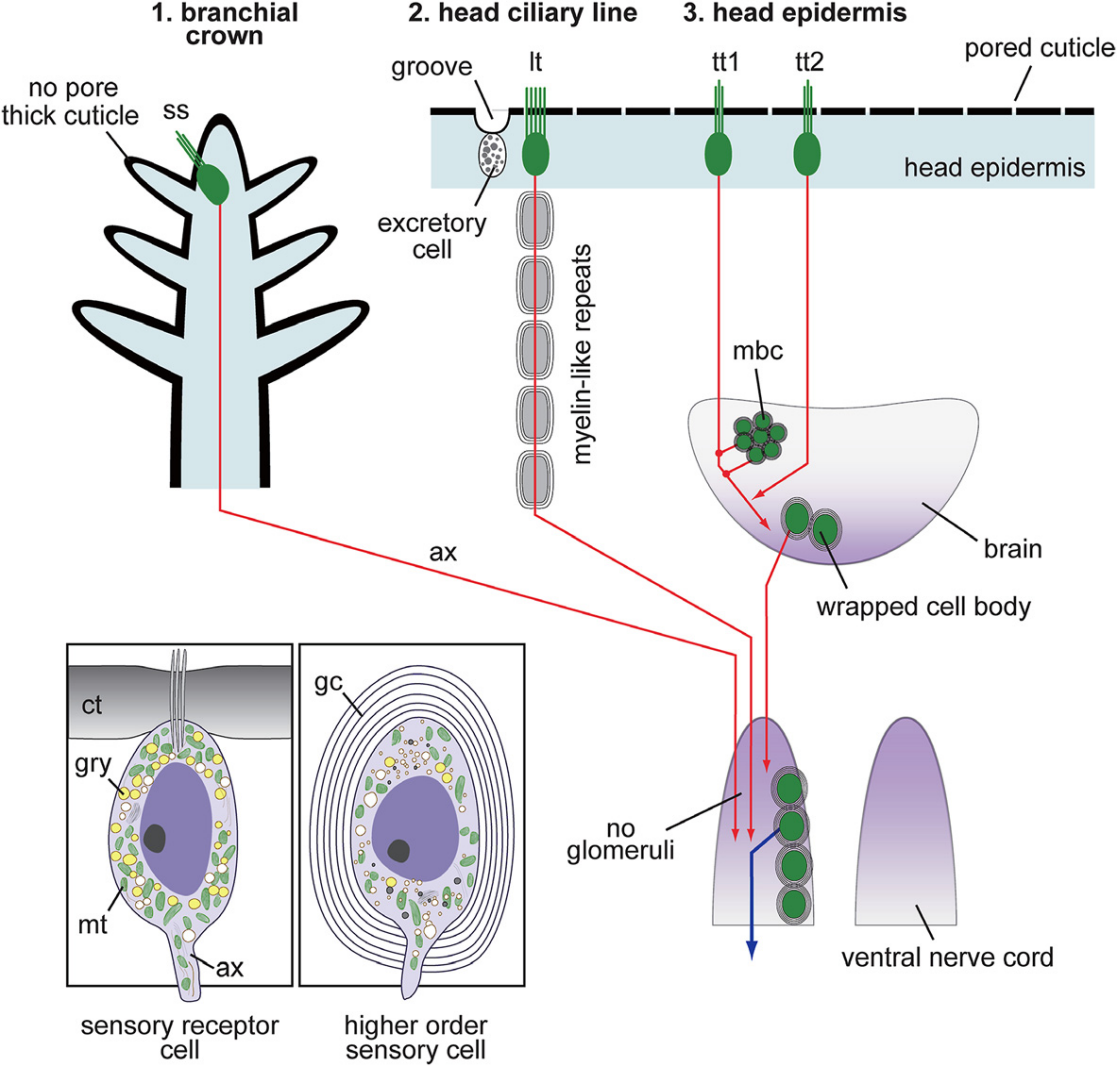
**Schemes of the sensory pathways and the differentially specialized protection systems.** In contrast to the primary sensory cells situated on the epidermis, the sensory neurons are multiply covered by glial wrappers. The myelin-like repeats are only formed in the head ciliary lines and not in the ventral nerve cords. Insets: the schemes show two distinct structural types of primary sensory receptor cells of epidermis and neuronal cells of the brain and ventral nerve cords. ax, axonal fiber; ct, cuticle; gc, glial cell membranes; gry, yellow color granule; lt, line type cell; mt, mitochondrion; ss, sensilla type cell; tt1 an tt2, tuft type cell 1 or 2.

We identified a total of four sensory cell types with possible homologies to other annelids based on the structure of the cilia, patterning of the cell clusters, and position on the body (Table 1). The forms of most alvinellid cells are rather simple, and do not display extreme modification such as the chambered or balloon-like multi-ciliary dendrites of the nuchal organs of lugworms [26]. The sensory receptor cells of alvinellid worms are generally found within the epithelium, and connect to the adjoining cells by the anchoring membrane junction. The alvinellid sensory cells are bipolar, with long axonal fibers, and penetrative cilia exhibiting 9 + 2 patterns of axonemal microtubules with additional microvilli, similar to those found in most polychaetes [26,28]. However, the most striking morphological features of the alvinellid sensory cells are as seen in Figure 4. 1) The thick outer cuticle, often without penetrative pores, in the branchial crown; 2) many yellow-colored and electron-dense granules distributed in the cells; 3) mitochondria-rich sensory cells (e.g., more than 50 non-branched mitochondria (n >6 cells) were observed in the single ultrathin section of the sensory cell, compared with less than five to ten mitochondria in the lugworm *Arenicola marina* [25]). Furthermore, there are few Golgi apparatus and endoplasmic reticulum compared with the ciliary sensory cells of lugworms [26]; and 4) the axons from the sensory cells are tightly covered with multi-layered myelinated glial membranes, although this varies among the different body parts and functional subsystems for chemical sensing (Figure 11).

### The glial membrane supporters

The types of glial systems identified in *P. hessleri* have not been described in any animals, except for a scale-worm also endemic to hydrothermal vents (Shigeno et al., submitted). The neural cells of many animals are more or less protected by glia, and the specialized myelinating glia for the long axons are found in many vertebrates, crustaceans, and annelids [46]. In the case of well-studied earthworms, the median and lateral giant axons of the ventral nerve cords are encompassed by concentrically wrapped lamellar sheets of insulating plasma membrane [47]. In this study, the glial system found in the alvinellid worms is considerably different. First, the myelin structure is present in the head sensory input systems and not found in the ventral nerve cords. Second, in addition to the axonal bundles, the cell bodies of the brain themselves are covered with multilamellar sheets.

The glial cells in vertebrates and some invertebrates serve diverse functions, including as a nutrition source, and for immunity, peptide signaling, neural development, as a resistor in fine electronic signal modifiers. They also provide protection by filtering or blocking toxic substances, including oxidative stressors and heavy metals, through maintenance of the chemical environment used to conduct electrical impulses [48-50]. If the cells are myelinating, the conduction speed of electrical impulses increases, thus providing an adaptive advantage for rapid behavioral responses to stimuli from alarm cues [51]. In the environment of hydrothermal vent fields, it is likely that specific glial protectors are required, since the environment is rich in heavy metals, hydrogen sulfide, and other toxic chemicals and metabolites produced by high temperature and pressure.

In addition, moderately hypoxic conditions are ubiquitous close to the vent field; therefore, some mechanism of protection is required to prevent the breakdown of glial membranes. This breakdown leads to irreversible cell death due to the production of reactive oxygen substances via hypoxia, which is well-studied in mammalian brains affected by ischemic stroke and integrity loss of the blood–brain barrier [52-54]. The actual functions of glial cells in alvinellids remain largely speculative, and it is not known how many glial cell types are present in these worms; however, our findings provide the first evidence for the role of such specialized glial systems in the hydrothermal endemic animals. Our findings also showed that not all of the sensory and neural cells are covered with multi-glial membranes, perhaps due to the presence of an alternative protector, such as the thick, non-pored collagenous walls in the epithelium of branches (summarized in Figure 11). This discovery suggests that unexpected biochemical heterogeneity and protection mechanisms may be present in the internal body spaces of these worms.

### Specialization of the central nervous system

**Figure 12:**
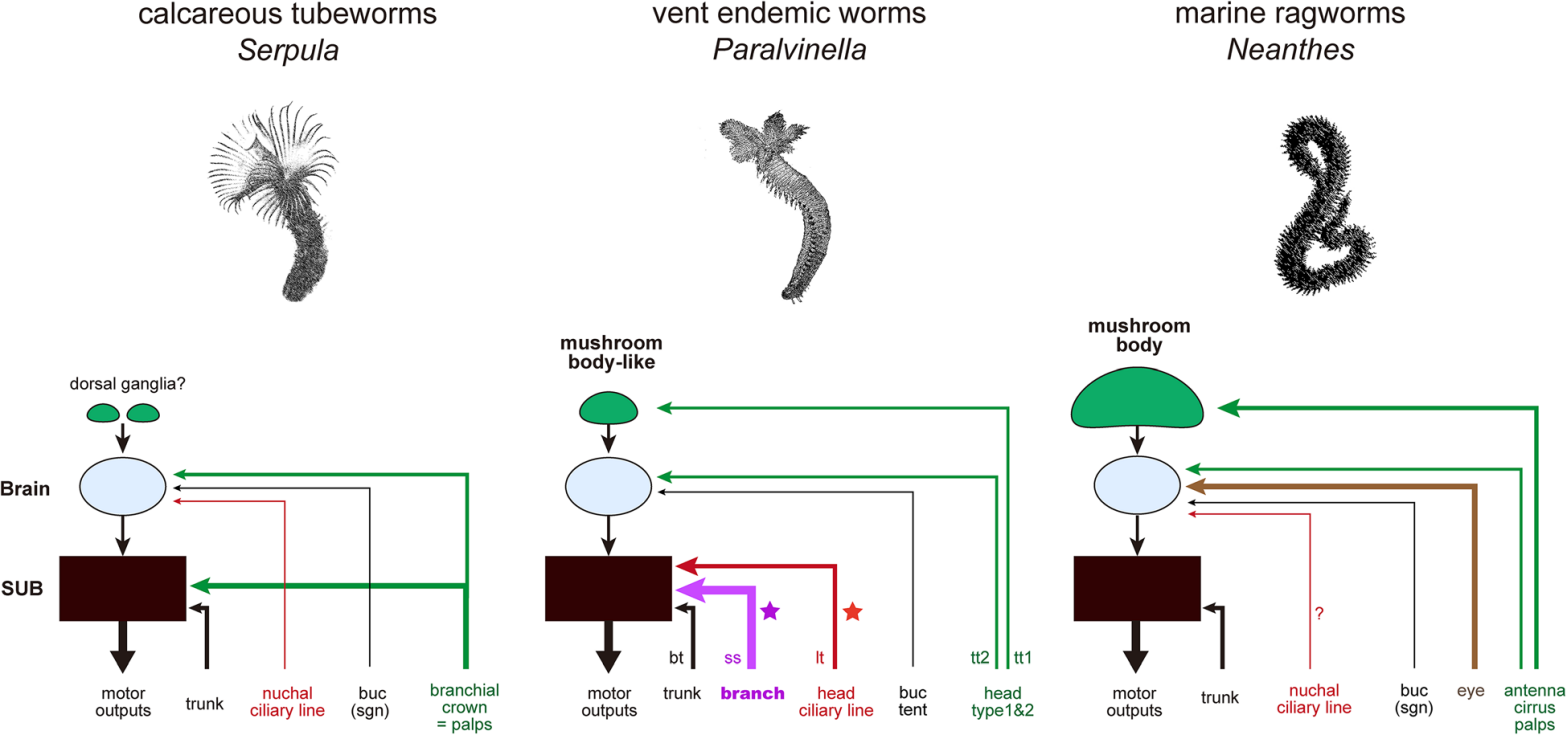
**Comparative schemes of the sensory inputs and motor outputs to emphasize the functional subdivisions.** The tuft type sensory cells and their inputs are characterized for alvinellid worms. The two distinct tuft type cells on the head or prostomium are identified. Note that the branchial crown of Terebellida (*Paralvinella*) is not homologous to those of Serpulidae and palps of Nereididae. Some neural pathways are not precisely identified. Data are simplified from Orrhage and Müller [30]. bt, bud type cell; lt, line type; ss, sensilla type; tt, tuft type; VNC, subesophageal ganglia with ventral nerve cord/ganglia.

Based on a comparative analysis of polychaete brains, the overall organization of the alvinellid brain can be compared to that of *Serpula vermicularis* and *Pista cristata*, both of which are burrowing species and use the numerous elongate branches or buccal tentacles for deposit feeding [30,55]. The brains of the alvinellids and these species are externally flat in shape, and are attached to the subesophageal mass and the ventral nerve cords with connective bundles. Many axonal tracts are broadly distributed without fasciculation in the neuropils, and in contrast with nereids and scale-worms, their compartments are not specialized [28,56]. Despite such similar basic morphology, the tract pathways related to the functional organization are dissimilar (Figure 12). First, the nerves from the branchial crown usually construct the major anterior components of the brain, as in the calcareous tubeworm *Serpula;* however, in alvinellids they run directly to the subesophageal mass or ventral nerve cords. Second, the nuchal organs are more centrally located and extend into the anterior region of the brain in *Serpula* and *Pista*, but in alvinellids the main pathways for the head line type cells are positioned similarly as in the nuchal organs, and run directly to the subesophageal mass or ventral nerve cords.

### The mushroom body-like structure

One of the most distinguished areas in the polychaete brains is the corpora pedunculata, also known as mushroom bodies, a pair of neuropils with associated small somata called globuli cells. These may function in the chemosensory learning and memory centers found in many polychaetes [28,42], but are reduced [31] or not developed in sessile species, including the calcareous tubeworm *Serpula* [30]. However, this does not mean that all sessile species lack mushroom bodies and globuli cells. In this study, we observed a pair of interneuron clusters in the anterolateral sides of the brain, indicating that these clusters might be positionally and functionally compared to the annelid mushroom bodies as homologous structures for the higher-order processing centers [31]. Alternatively, higher-order neurons are often developed as distinct interneurons for a type of chemosensory system such as in the brains of aplacophoran molluscs [57,58]. This indicates that the interneuron pools of alvinellids may have developed independently to act as specialized sensory receptors unique to hydrothermal vents.

One could assume that the highly developed branchial crown functions as a sensory organ due to the direct exposure of the bacteria-covered tubes to hot vent fluids. In this study, most sensory cells in the branches were of the ciliate type, and long axons extend into the subesophageal mass or the ventral nerve cords where there were no clearly identifiable lobes or neuropil compartments. Additionally, the buccal tentacles may possibly be used for the bacterial feeding (Shigeno, unpublished data), and have two sensory ciliated cell types and specific neuropil compartments that were not observed in the brain. If specialization of the neuropil compartments of chemosensory or gustatory centers are related to their chemical receptor diversity, as in the case of the glomeruli centers in annelid brains [31], we may expect that the sensing capacity of the branchial crown and buccal tentacles of the alvinellid worms is less specialized, and chemicals detected are not processed as environmental information. Whether or not the chemical receptors of alvinellids are specialized remains to be determined, and continued molecular studies of ionotropic, gustatory, and olfactory receptors are needed to better understand the chemosensory systems of animals endemic to hydrothermal vents.

### Comparative evolutionary frameworks

As in free-living marine ragworms and sessile calcareous tubeworms, the sensory information collected by alvinellid sensory cells is processed by the mushroom bodies or comparable higher-order sensory centers, or the brain centers or the ventral nerve cords, as illustrated in Figure 11. This scheme for the sensory input and output signaling emphasizes the specific characteristics of alvinellid sensory processing systems.

First, the axonal projections of the line type sensory cells or the nuchal organ of *P. hessleri* extend directly into the subesophageal ganglia. This situation is dissimilar to that of the calcareous tubeworm *Serpula,* where the nuchal pathways extend into the brain (Figure 11; [59]). Additionally, the nuchal organ related centers of alvinellids are less specialized than those of the bloodworm *Glycera rouxii,* which has distinct centers known as annexed ganglia and associated giant cells [30], suggesting that the alvinellid cilliary cells might have a simple receptor capacity. Second, the signals from the branchial crown of *P. hessleri* are processed only in the subesophageal ganglia, whereas the sensory cells of the branchial crown in calcareous tubeworms project into both the brain and subesophageal ganglia [59]. Third, the mushroom bodies are composed of small interneurons and as in the ragworms, are located at the antero-dorsal sides of the brain [31]; however, the alvinellid mushroom bodies receive inputs from the broadly distributed single sensory cell types of the head (see Figure 11). In addition, the alvinellid sensory inputs to the brain through the mushroom bodies are similar to those of free-living ragworms in the genus *Neanthes* [30]. We therefore propose that the input systems from the eyes, antenna, cirrus, and pulps of the ragworms, which are not developed in the alvinellid head parts, could be compared to those of the type 1 and type 2 sensory cells. We further suggest that alvinellids utilize two distinct sub-systems for sensory signaling: (1) the brain, which serves as a primary sensory processing system, and (2) several “short-cut” pathways from the head and branchial sensory cells to the subesophageal and ventral nerve cords, presumably for the simple and rapid transduction of environmental sensory signals to the trunk motor control networks or any adaptive organs, which regulate homeostasis through the endocrine and circulatory systems in the trunk region.

## Materials and methods

### Animal samples

More than three hundred individuals of adults or juveniles of alvinellids, *Paralvinella hessleri* [60], Polychaeta, Sedentaria, and Alvinellidae, were collected aboard the research vessel “Natsushima” during research cruise NT12-10 (31°53.049′ N, 139°58.104′ E, 907 m depth, off Myojin-sho submarine caldera, onboard ID 1374–9; or 32°06.214′ N, 139°52.05′ E, 1294 m depth, off Myojin Knoll, onboard ID 1377–4), in the Izu-Ogasawara Arc, Japan. Samples were collected on the 25th or 29th of April 2012 with Hyper-Dolphin 3000 remotely operated vehicles (HPD#1374 or #1377). Worms were collected using a suction sampler from the surface of the white microbial mat with worm’s nests attached to the chimney walls. The individuals used in this study were fresh and active animals, which were selected by eye for collection. Following the ROV field surveys, the live animals were maintained in non-aerated cold deep-sea water collected in the same canisters, and specimens were stored at 4–8°C for a few days.

### Histology and immunocytochemistry

The specimens were fixed on board with 4% paraformaldehyde in phosphate buffered saline (PBS); alternatively, the suction sampler and canister were filled with deep-sea water (4°C) for 12 hours, then were washed in PBS, and transferred to 80% methanol or ethanol for long term storage at −30°C or −80°C. Immunostaining of the whole-mounts followed a standard protocol (n >30). The whole-mounts were treated with Proteinase K (5 μg/ml in PBS) for 5 min at 37°C. A mouse monoclonal anti-acetylated alpha-tubulin antibody (6-11B-1 clone) isolated following immunization with sea urchin flagella proteins (acTUBA, Sigma Chemical, T6793, 1:3000) in PBST (PBS with 1% Tween 20 and 1% BSA), or rabbit polyclonal anti-serotonin (5-hydroxytryptamine, 5-HT, 1:500) antibody (Sigma, S5545, 1:500) was used to detect 5-HT positive selected neurons. These primary antibodies have been widely employed in invertebrate neuroanatomy (e.g., [41]). A CF™ 488A goat anti-mouse IgG or CF™ 594 goat anti-rabbit IgG antibody (Biotium Incorporated, 1:400) was used as a secondary antibody. DAPI (4′-6-Diamidino-2-phenylindole; Sigma, 0.1μg/ml), SYTO®13, a green-fluorescent nucleic acid stain dye (Invitrogen, 1:5000), and rhodamine-conjugated Concanavalin A (ConA, Vector Laboratories, 1:1000) and CF™ 594 phalloidin (Cytoskeleton Incorporated, 1:600) were used for counterstaining of the nuclei, the cell membrane, or F-actin rich muscle fibers, respectively. Samples were examined using confocal laser scanning microscopy as described below. Some paraformaldehyde fixed samples were embedded in paraffin and cut with a rotatory microtome to a 5–10 μm thickness. These sections were stained in Mayer’s hematoxylin and eosin solution.

### Electron microscopy ultrastructural analysis

The electron microscopy analyses were conducted according to previously described methods [61], with some modifications. The whole-mount or dissected specimens (n >10) were fixed in a solution of 2.5% glutaraldehyde in cold deep-sea water for at least one week. After extensive washes with 0.22 mm-filtered seawater, samples were postfixed with 2% osmium tetroxide in filtered seawater for two hours at 4°C. For field-emission scanning microscopy (FE-SEM), samples were stained with 1% aqueous tannic acid (pH 6.8) for one hour and treated with 1% aqueous osmium tetroxide for one hour at 4°C. Samples were dehydrated in a graded ethanol series, critical point-dried (JCPD-5; JEOL Ltd., Tokyo, Japan), coated using an osmium plasma coater (POC-3; Meiwa Shoji Co., Osaka, Japan), and observed under an FE-SEM (JSM-6700F; JEOL Ltd., Tokyo, Japan). For transmission electron microscopy (TEM), the postfixed animals were rinsed with distilled water, and stained with 1% aqueous uranyl acetate for two hours at 4°C. The samples were then rinsed, dehydrated, and embedded in Epon 812 resin (TAAB, Aldermaston, UK). Ultrathin sections were obtained with an ultramicrotome (thickness 60nm; Reichert Ultracut S; Leica, Wetzlar, Germany), and the sections were double stained with uranyl acetate and lead citrate for observation under the TEM (Tecnai 20; FEI company, Tokyo, Japan) operated at 120 kV. The photos were taken with resolution 2048 × 2115 pixel.

### Neurotracing analysis

The lipophilic dye tracers NeuroVue® maroon or red (Polysciences Incorporated, Warrington, PA, USA) were used for neuronal tract tracing according to the manufacturer’s protocol, with some modifications. The dyes transfer into plasma membranes in formaldehyde fixed tissues and diffuses laterally within the membrane, finally labeling the cell bodies, axons, and allowing visualization of neuronal processes. More than twenty worms were fixed in 4% paraformaldehyde in cold deep-sea water overnight, and washed in PBS without any polysorbate detergents and alcohol. The fine pieces of coated dye filters were directly applied to the head part with the fine tips of capillary tubes, and they were stored in 0.1% paraformaldehyde in PBS, and at 37°C for one to three weeks. The dye tracers transferred into the epidermal tissues and diffused in the neuronal membranes, allowing visualization of the selected or accidentally labeled cell bodies and long axonal arborization. ConA and Alexa Fluor® 350 or 594 were used as a versatile lectin probe for the membrane counterstaining to illuminate dye-traced cells. The whole-mount samples were stained with other dyes and viewed using confocal microscopy as described below.

### Image processing

Samples were examined as whole-mounts or sections using the confocal laser scanning microscope Fluoview, FV500 ver4.3c (Olympus, Lake Success, NY, USA) with the fluorescent microscope IX-71 automated inverted microscope platform (Olympus). Laser power was employed with UV, Argon, and HeNe, and the appropriate filter set was selected according to the fluorescent markers. The pseudo-colors were used to enhance the structural images according to the manufacturer’s protocol. Additional processing of images for contrast, brightness, and color balance was performed as needed with Adobe Photoshop CS5 (Adobe Systems Incorporated, San Jose, CA, USA). Schematic diagrams were created with Adobe Illustrator CS5 (Adobe Systems Incorporated).

### Terminology

The terms used for the alvinellid sensory cells and nervous systems are based on the definitions given by previous studies [29,37,38], unless otherwise specified. Richter et al. [62] was also used as a nomenclatural reference for invertebrate neuroanatomical terms. Desbruyeres et al. [2] was used for alvinellid anatomical terms.

## Additional files

Additional file 1: Video S1. A movie of whole-mount confocal Z-projection image stacks of a left head part of *Paralvinella hessleri*. See Figure 5D-H for details. Format: MP4 Size: 921 KB. The video can be viewed with Quick Time Player, Apple Inc. Mac OSX, ver.10.3. Additional file 2: Video S2. A movie of whole-mount confocal Z-projection image stacks of a right side of the brain of *Paralvinella hessleri.* See Figure 7E-I for details. Format: MP4 Size: 1.3 MB. Additional file 3: Video S3. A movie of whole-mount confocal Z-projection image stacks of a part of branchial crown of *Paralvinella hessleri.* See Figure 9D and E for details. Format: MP4 Size: 954 KB. Additional file 4: Video S4. A movie of whole-mount confocal Z-projection image stacks of a part of the notopodia and sensilla of *Paralvinella hessleri.* See text and Figure 10B for details. Format: MP4 Size: 1.4 MB. Additional file 5: Figure S1. Details of the trunk and ciliary types. A. Side view of the trunk showing the distribution of bud type (arrowheads) and possible developing or different type of tuft type cilia (arrows), a SEM image. B. Enlarged view of tuft type cilia (arrows) with the ventral patch type. C. A confocal optical section of the trunk, ventral view. The acetylated alpha-tubulin (acTUBA) positive cilia are seen. The DAPI (nuclei, blue) and phalloidin (actin, red) cell membrane markers used for counterstaining. D and E. Enlarged views of a part of the C showing the ventral patch type cilia and possible tuft types (arrows). vpt, ventral patch type cilia. Scale bar in A, C: 50 μm; B, D, E: 10 μm.
