# Supplementary figures and images for: Sensing deep extreme environments: the receptor cell types, brain centers, and multi-layer neural packaging of hydrothermal vent endemic worms
Source: Front Zool. 2014 Nov 18;11:82. doi: 10.1186/s12983-014-0082-9 (PMC4261566; doi:10.1186/s12983-014-0082-9)

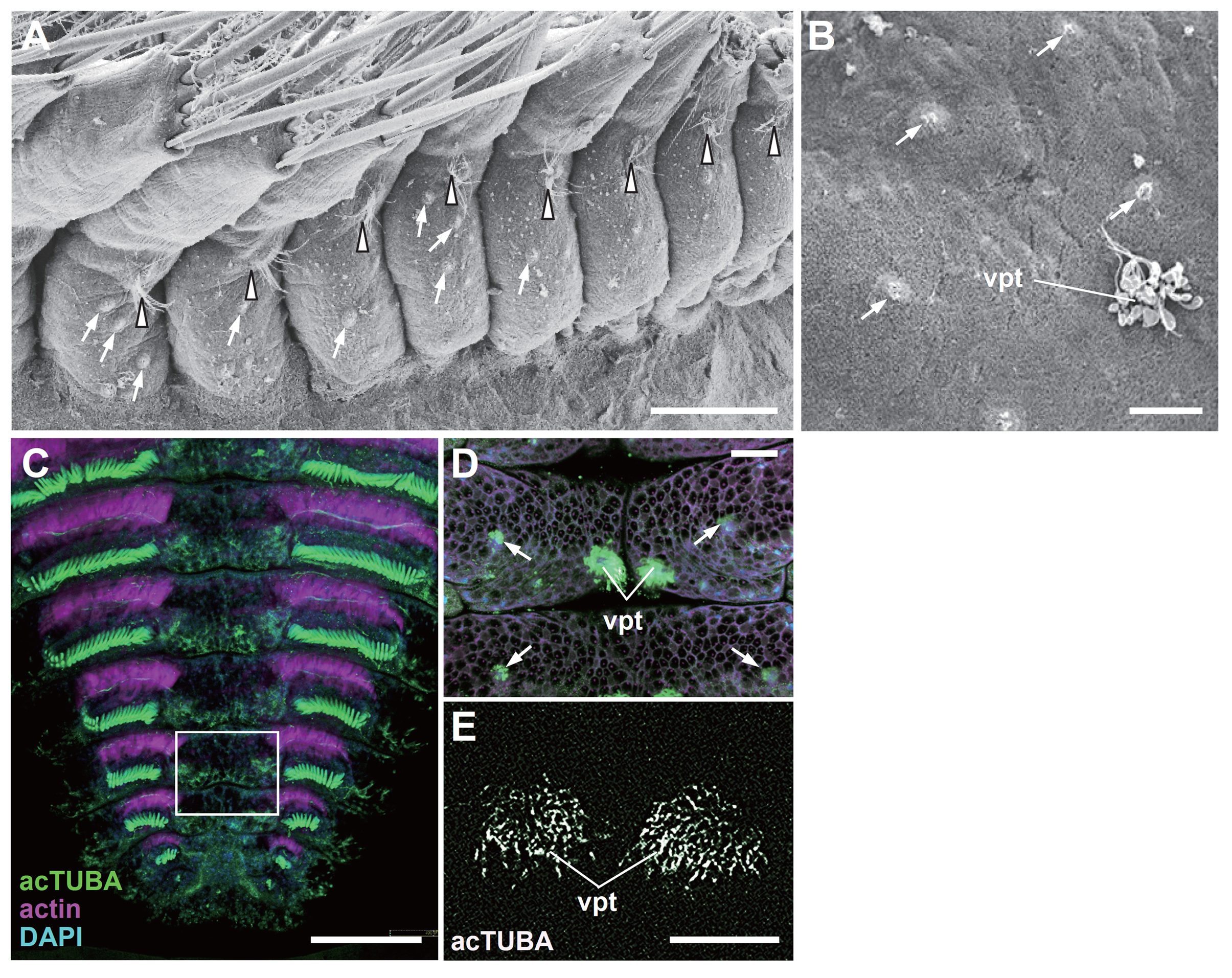

Supplement: Additional file 5: Figure S1. — Details of the trunk and ciliary types. A. Side view of the trunk showing the distribution of bud type (arrowheads) and possible developing or different type of tuft type cilia (arrows), a SEM image. B. Enlarged view of tuft type cilia (arrows) with the ventral patch type. C. A confocal optical section of the trunk, ventral view. The acetylated alpha-tubulin (acTUBA) positive cilia are seen. The DAPI (nuclei, blue) and phalloidin (actin, red) cell membrane markers used for counterstaining. D and E. Enlarged views of a part of the C showing the ventral patch type cilia and possible tuft types (arrows). vpt, ventral patch type cilia. Scale bar in A, C: 50 μm; B, D, E: 10 μm. [file 12983_2014_82_MOESM5_ESM.tiff]
